# Novel roles of the multi-functional CCR4-NOT complex in post-transcriptional regulation

**DOI:** 10.3389/fgene.2014.00135

**Published:** 2014-05-20

**Authors:** Toshifumi Inada, Shiho Makino

**Affiliations:** Laboratory of Gene Regulation, Graduate School of Pharmaceutical Sciences, Tohoku UniversitySendai, Japan

**Keywords:** translation, deadenylation, miRNA, mRNA stability, protein quality control, ubiquitin-protein ligases, proteasome, decapping

## Abstract

The CCR4-NOT complex is a highly conserved specific gene silencer that also serves more general post-transcriptional functions. Specific regulatory proteins including the miRNA-induced silencing complex and its associated proteins, bind to 3’-UTR elements of mRNA and recruit the CCR4-NOT complex thereby promoting poly(A) shortening and repressing translation and/or mRNA degradation. Recent studies have shown that the CCR4-NOT complex that is tethered to mRNA by such regulator(s) represses translation and facilitates mRNA decay independent of a poly(A) tail and its shortening. In addition to deadenylase activity, the CCR4-NOT complex also has an E3 ubiquitin ligase activity and is involved in a novel protein quality control system, i.e., co-translational proteasomal-degradation of aberrant proteins. In this review, we describe recent progress in elucidation of novel roles of the multi-functional complex CCR4-NOT in post-transcriptional regulation.

## INTRODUCTION

CCR4-NOT is a highly conserved multiprotein complex that plays various roles in gene regulation. In the nucleus, this complex is involved in chromatin modifications, transcription elongation, RNA export, nuclear RNA surveillance, and DNA repair coupled with transcription (Collart and Panasenko, 2012; Miller and Reese, 2012; Wahle and Winkler, 2013). In the cytoplasm, the CCR4-NOT complex is the main deadenylase and plays crucial roles in mRNA decay and translation repression induced by poly(A) shortening (Wahle and Winkler, 2013). The CCR4-NOT complex also plays central roles in post-transcriptional regulation including translation repression independent of a poly(A) tail (Cooke et al., 2010). CCR4-NOT has an E3 ubiquitin ligase activity as well as a deadenylase activity, and plays important roles in co-translational protein quality control (Collart and Panasenko, 2012). This review focuses on novel functional aspects of the CCR4-NOT complex in post-transcriptional regulation and provides insight into the mechanism of gene regulation by this conserved multi-functional complex and its associated regulators.

## THE CCR4-NOT COMPLEX AS THE MAIN CELLULAR DEADENYLASE

### DEADENYLATION AS A KEY STEP FOR mRNA DEGRADATION

Deadenylation is a key step in the degradation of most mRNAs (Coller and Parker, 2004; Parker and Song, 2004; Parker, 2012). CCR4-NOT4 contributes to the initial step of 3′-poly(A) tail shortening that triggers the removal of the cap structure by recruitment of the decapping complex. Thus deadenylation results in reduced binding of the poly(A) binding protein to the poly(A) tail leading to a reduced rate of translation initiation. Translation slow-down results in recruitment of the decapping complex. Subsequent removal of the cap structure leads to rapid degradation of the body of the mRNA by Xrn1, a conserved 5′-to-3′ exoribonuclease. PAN2-PAN3 is also an important cellular deadenylase and there is significant redundancy and functional overlap between PAN2-PAN3 and CCR4 deadenylases (Parker and Song, 2004; Wahle and Winkler, 2013; Wolf and Passmore, 2014). An alternative pathway of degradation of the mRNA body is through 3′-to-5′ exoribonucleolytic degradation by the exosome complex in association with the Ski complex (Anderson and Parker, 1998). The yeast Ski complex is a tetramer composed of the DEVH ATPase Ski2, Ski3, and Ski8 in a 1:1:2 ratio, and forms a joint substrate channel with the exosome that shunts RNA from the helicase to the nuclease activity (Halbach et al., 2013).

### CATALYTIC SUBUNITS AND STRUCTURE OF CCR4-NOT

The yeast CCR4-NOT is a large multiprotein complex with an approximate mass of 1 MDa, is consisted of CCR4 and three CAF proteins and five NOT proteins (Bai et al., 1999; Maillet et al., 2000). Two subunits of the yeast CCR4-NOT complex, a 96 kDa CCR4 subunit (CNOT6/6L in vertebrates) and a 50 kDa CAF1/POP2 subunit (CNOT7/8 in vertebrates), have nuclease activity. CCR4 is an endonuclease-exonuclease-phosphatase (EEP) superfamily protein and contains a DNase I-like domain. CAF1 is a DEDD (Asp-Glu-Asp-Asp) family protein and contains an RNase D-like domain. Both CCR4 and CAF1 are necessary for deadenylase activity in yeast, and the catalytic activity of CCR4 contributes to this deadenylase activity (Chen et al., 2002; Tucker et al., 2002; Viswanathan et al., 2003; Goldstrohm et al., 2007). The CAF1 orthologs, CNOT7 and CNOT8, in human cells, trypanosomes and *Drosophila*, also appear to function as the major deadenylase for mRNA degradation (Temme et al., 2004; Viswanathan et al., 2004; Schwede et al., 2008).

Structural studies show that the N-terminal arm of NOT1, another 240 kDa subunit of yeast CCR4-NOT, has a HEAT-repeat structure with domains related to the MIF4G fold (Bartlam and Yamamoto, 2010; Basquin et al., 2012; Petit et al., 2012). The MIF4G domain of NOT1 recognizes CAF1, which, in turn, binds the LRR domain of CCR4 and tethers the CCR4 nuclease domain. Thus the NOT1 subunit places the two nucleases CCR4 and CAF1/POP2 in a pivotal position within the CCR4-NOT complex. Their specific disruption affects both cell growth and mRNA deadenylation and decay *in vivo* in yeast (Basquin et al., 2012). The crystal structure of the human NOT module formed by the C-terminal region of CNOT1 with the C-terminal regions of two other subunits of the CCR4-NOT complex, CNOT2 and CNOT3, clearly shows that the CNOT1 C-terminal region provides a rigid scaffold consisting of two perpendicular stacks of HEAT-like repeats (Bhaskar et al., 2013; Boland et al., 2013). The heterodimer of CNOT2 and CNOT3 is tightly associated with the surface of CNOT1. These data suggest that the scaffolding function of NOT1 within the CCR4-NOT complex is evolutionarily conserved (Bhaskar et al., 2013; Boland et al., 2013).

## RECRUITMENT OF CCR4-NOT BY REGULATOR PROTEINS

**FIGURE 1:**
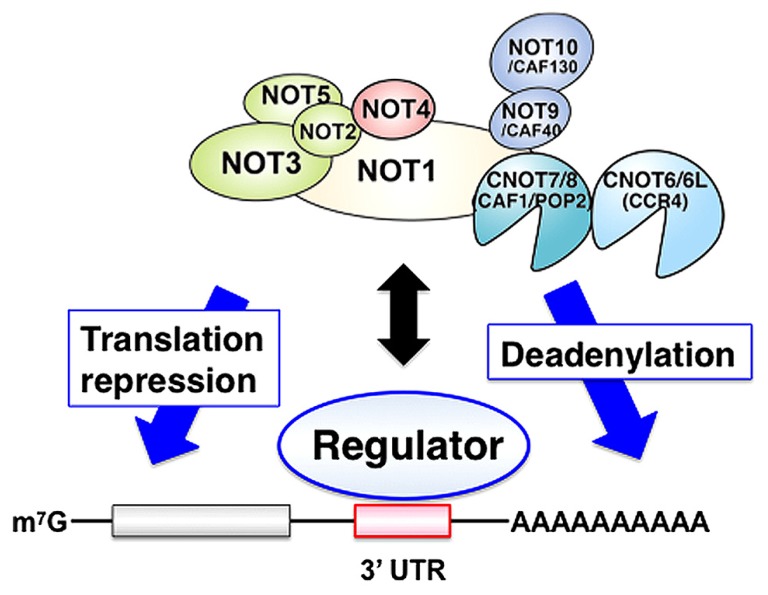
**The CCR4-NOT complex regulates specific mRNAs at the post-transcriptional level**. Specific regulators directly bind to the 3′ untranslated region (UTR) of target mRNAs and recruit the CCR4-NOT complex to these target mRNAs thereby stimulating mRNA decay and/or repressing translation.

The CCR4-NOT complex regulates specific mRNAs following its recruitment to target mRNAs by regulator proteins. Thus, RNA binding proteins directly bind to the 3′ untranslated region (UTR) of target mRNAs and recruit the CCR4-NOT complex thereby stimulating mRNA decay or repressing translation (**Figure 1**).

### TRISTETRAPROLIN (TTP)

Tristetraprolin is an RNA binding protein that mediates the rapid degradation of mRNAs containing AU-rich elements (AREs). CCR4-NOT is recruited to mRNAs targeted for degradation by TTP (Sandler et al., 2011; Fabian et al., 2013) via NOT1, which is the largest subunit of the CCR4-NOT complex and serves as a scaffold for other subunits of the complex. In the cytoplasm, human NOT1 interacts with both the C-terminal domain of TPP and with CAF1 (Fabian et al., 2013). Furthermore, NOT1 is required for the rapid decay of ARE-mRNAs, and TTP can only recruit CCR4-NOT in the presence of NOT1 (Sandler and Stoecklin, 2008; Sandler et al., 2011). Thus, cytoplasmic NOT1 mediates recruitment of the CNOT7(CAF1/POP2) and CNOT6(CCR4) deadenylases by a specific RNA binding protein thereby triggering the decay of its target mRNAs. TTP is an important regulator of the inflammatory response and acts as a bona fide tumor suppressor in lymphomas by binding to the AREs of Interleukin mRNAs and recruiting the CCR4-NOT complex to promote mRNA deadenylation (Ross et al., 2012; Sanduja et al., 2012).

### PUMILIO AND NANOS FAMILY PROTEINS

In yeast, Mpt5, a Pumilio family protein, binds to the 3′ UTR of *HO* mRNA, a DNA endonuclease for mating-type switching. Mpt5p recruits the CAF1 subunit of the CCR4-NOT complex and promotes deadenylation of the target *HO* mRNA (Goldstrohm et al., 2006, 2007). Pumilio proteins also bind Caf1 in humans, *Caenorhabditis elegans* and *Drosophila* (Goldstrohm et al., 2006; Kadyrova et al., 2007). In *Drosophila*, Pumilo and Nanos bind to Cyclin B and *hunchback* mRNA, and recruit the CCR4-NOT complex (Kadyrova et al., 2007). Pumilio binds to CAF1/POP2 and Nanos binds to CCR4 (Kadyrova et al., 2007; Joly et al., 2013). Pumilio and Nanos also interact with each other.

Nanos is one of a number of evolutionarily conserved proteins implicated in germ cell development (Tsuda et al., 2003). NANOS2 plays an important role in both the maintenance and sexual development of germ cells (Suzuki et al., 2007; Saga, 2008a, b, 2010). NANOS2 interacts with the CCR4-NOT deadenylation complex leading to suppression of specific RNAs. The complex containing NANOS2 and CCR4-NOT has deadenylase activity *in vitro*. Moreover, some of the RNAs implicated in meiosis interact with NANOS2 and accumulate in its absence (Suzuki et al., 2010, 2012). NANOS2 localizes to P-bodies, to which CNOT proteins have been localized *in vivo* (Suzuki et al., 2010, 2012).

## THE ROLE OF CCR4-NOT IN mRNA SURVEILLANCE SYSTEMS

The CCR4-NOT complex plays crucial roles in nuclear RNA surveillance (Azzouz et al., 2009a, b). Aberrant polyadenylated snoRNAs and rRNAs are usually degraded by the nuclear surveillance machinery that is composed of the exosome and its nuclear co-factor TRAMP (Houseley and Tollervey, 2008). These aberrant RNAs are accumulated in yeast mutants of the CCR4-NOT complex, and the CCR4-NOT complex interacts with TRAMP subunits or with the exosome (Azzouz et al., 2009a). Therefore, interaction between the CCR4-NOT complex and the nuclear exosome may be required for appropriate nuclear exosome function *in vivo* (Vanacova and Stefl, 2007). In humans, an unconventional CCR4-CAF1 complex accumulates in Cajal bodies, sub-nuclear foci thought to be sites where snRNA and snoRNA mature (Wagner et al., 2007). The function of the deadenylase complexes CCR4-NOT and CCR4-CAF1 in nuclear mRNA surveillance is likely to be conserved.

The CCR4-NOT complex also plays crucial role in the cytoplasmic mRNA surveillance system (Popp and Maquat, 2013). Nonsense-mediated mRNA decay (NMD) is a eukaryotic quality control mechanism that detects aberrant mRNAs containing a premature termination codon and induces their rapid degradation (Yamashita et al., 2005; Nicholson and Muhlemann, 2010; Popp and Maquat, 2013; Schweingruber et al., 2013). In mammalian cells, the rapid degradation of NMD target mRNAs is mediated by SMG5 and SMG7 proteins, which recruit general mRNA decay enzymes. The SMG5–SMG7 heterodimer directly recruits the CCR4-NOT complex to mRNAs containing a premature termination codon via interaction with CAF1/POP2 and elicits deadenylation-dependent decapping and rapid decay of the body of NMD target mRNAs (Loh et al., 2013). SMG6, an NMD specific endonuclease, also plays a crucial role in the degradation of aberrant mRNA containing a premature termination codon in both *Drosophila* (Gatfield and Izaurralde, 2004; Kashima et al., 2010) and human cells (Eberle et al., 2009; Loh et al., 2013). The SMG7-CAF1/POP2 interaction is critical for NMD in cells depleted of SMG6, indicating that SMG7 and SMG6 act redundantly to promote the degradation of NMD targets (Loh et al., 2013).

## FUNCTIONS OF THE CCR4-NOT COMPLEX IN GENE SILENCING BY miRNA

**FIGURE 2:**
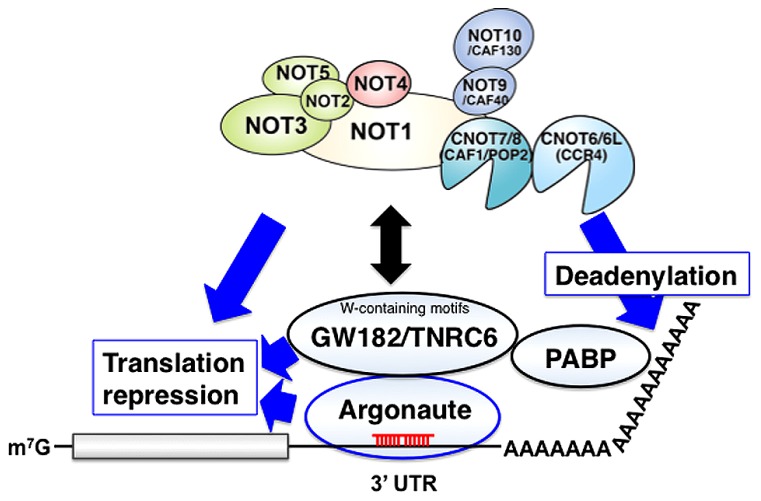
**The CCR4-NOT complex functions in gene silencing by miRNA**. MicroRNA (miRNA)-induced silencing complexes (miRISCs) on target mRNA contain both miRNA (red) and an Argonaute protein. The Argonaute protein interacts with a GW182/TNRC6 family protein, and these factors play crucial roles in miRNA-induced gene silencing including deadenylation, translational repression and the rapid decay of mRNA targets. Potential roles of CCR4-NOT in miRISCs: **Deadenylation:** GW182/TNRC6-mediated recruitment of CCR4-NOT requires conserved W-containing motifs of GW182/TNRC6, and C-terminal effector domain of GW182/TNRC6 is sufficient to bind CCR4-NOT and promote deadenylation of target mRNAs. Association of the CCR4-NOT complex with the GW182/TNRC6 protein releases PABP from the mRNA poly(A) tail, thereby disrupting mRNA circularization and facilitating translational repression and deadenylation. **Translation repression:** MicroRNA-induced translation repression occurs independent of deadenylation by CCR4-NOT in various organisms. The molecular mechanism by which miRISC inhibits translation is still largely unknown. **Decapping:** miRISC also triggers decapping and the subsequent degradation of mRNA targets independently of ongoing deadenylation. CCR4-NOT may be involved in this regulation.

MicroRNA (miRNA)-induced silencing complexes (miRISCs) repress translation and promotes the degradation of miRNA targets. GW182/TNRC6 family proteins interact with Argonaute (Ago) proteins and are required for the translational repression, deadenylation, and decay of miRNA targets (Braun et al., 2011, 2013; Chekulaeva et al., 2011; Fabian et al., 2011). *Drosophila* GW182 and its vertebrate ortholog TNRC6A-C are Ago-binding proteins that provide a platform for interaction with proteins such as poly(A) binding protein (PABP), PAN2-PAN3 and the CNOT1 subunit of the CCR4-NOT complex that regulate cellular RNA. PABP and PAN2-PAN3 appear to have auxiliary roles in miRNA-mediated silencing by augmenting miRISC binding to target mRNAs and potentiating miRNA-mediated deadenylation. In contrast, the interaction between GW182/TNRC6 and the CCR4-NOT complex appears to have indispensable roles in miRNA-mediated silencing (**Figure 2**).

### THE CCR4-NOT COMPLEX FACILITATES DEADENYLATION BY miRNA

Prior to degradation by the major cytoplasmic 5′-to-3′ exonuclease XRN1, mRNAs are first deadenylated and then decapped (Coller and Parker, 2004; Parker, 2012). mRNA deadenylation is catalyzed by the sequential action of two cytoplasmic deadenylase complexes, the PAN2-PAN3 and the CCR4-NOT complexes (Coller and Parker, 2004; Parker, 2012). CCR4-NOT deadenylates mRNA associated with miRNA-induced silencing complexes in human cells (Piao et al., 2010). GW182 proteins are recruited to the miRNA repression complex through direct interaction with Argonaute proteins, and represses target mRNA (Eulalio et al., 2008, 2009). In human and *Drosophila melanogaster* cells, gene silencing by miRNA involves GW182/TNRC6-mediated recruitment of CCR4-NOT through conserved W-containing motifs, Gly/Ser/Thr-Trp (G/S/TW) or Trp-Gly/Ser/Thr (WG/S/T), that are located in both the N-terminal and C-terminal effector domains of GW182/TNRC6 proteins (Chekulaeva et al., 2011; Fabian et al., 2011). Although G/S/TW or WG/S/T motif of GW182/TNRC6 is sufficient to bind CCR4-NOT, only one of these motifs can promote processive deadenylation of target mRNAs. In humans, TNRC6C also independently interacts with the PAN2-PAN3 complex and contributes to gene silencing by miRNA (Fabian et al., 2011; Christie et al., 2013).

### THE ROLE OF THE CCR4-NOT COMPLEX IN mRNA DECAPPING INDUCED BY miRNA

mRNA degradation by miRNAs and GW182/TNRC6 requires both the CCR4-NOT deadenylase and the DCP1-DCP2 decapping complexes (Rehwinkel et al., 2005; Behm-Ansmant et al., 2006). Decapping is catalyzed by DCP2, and the full activity or stability of DCP2 requires the additional decapping activators DCP1, HPat, EDC4 and the DEAD-box protein Me31B/DDX6/RCK/p54 (Tritschler et al., 2008, 2009; Haas et al., 2010). In yeast, CAF1/POP2 associates with DHH1, (an ortholog of DDX6/RCK/p54), functions in mRNA decapping and interacts with the decapping complexes (Coller et al., 2001; Coller and Parker, 2005; Nissan et al., 2010). Deadenylated miRNA target mRNAs accumulate when decapping is blocked, and miRISC enhances association of the target mRNAs with decapping activators, indicating a role for decapping activators in miRNA-mediated mRNA destabilization (Bagga et al., 2005; Behm-Ansmant et al., 2006; Rehwinkel et al., 2006). The recruitment of DCP1 and Me31B by miRISC occurs before the completion of deadenylation. DCP1 and Me31B can also be recruited by miRISC onto engineered miRNA target RNAs which lack a cap structure, a protein-coding region and a poly(A) tail (Nishihara et al., 2013). Therefore, miRISC can trigger decapping and the subsequent degradation of mRNA targets independently of ongoing deadenylation. Consistent with these data, recruitment of the NOT1 protein precedes the interaction of the decapping activator HPat with the miRNA effector complex in *Drosophila* (Barisic-Jager et al., 2013). This observation supports the proposal that HPat may couple deadenylation and decapping of target mRNA induced by miRNA (Haas et al., 2010).

### CCR4-NOT IS INVOLVED IN miRNA-INDUCED TRANSLATION REPRESSION

GW182 proteins interact with the PABP and the CCR4-NOT deadenylase complex, and facilitate miRNA target deadenylation. However, how GW182 proteins repress translation is still largely unknown. An recent study showed that the CCR4-NOT complex recruited by the GW182 protein releases PABP from the poly(A) tail, thereby disrupting mRNA circularization and facilitating translational repression and deadenylation (Zekri et al., 2013). GW182 proteins decrease the association of eIF4E, eIF4G and PABP with miRNA targets (Zekri et al., 2013). PABP dissociation requires interaction of GW182 proteins with the CCR4-NOT complex, and PABP dissociates from silenced targets in the absence of deadenylation (Zekri et al., 2013).

MicroRNA-induced translation repression occurs independent of deadenylation by CCR4-NOT in various organisms (Fukaya and Tomari, 2012; Mishima et al., 2012). Although *Xenopus* CCR4 and CAF1/POP2 enzymes are active deadenylases, tethering of CCR4 to target mRNA represses translation independent of deadenylation (Cooke et al., 2010). A recent study proposed that translational repression and eIF4AII activity are critical for miRNA-mediated gene silencing and that miRISC inhibits ribosome scanning by recruiting the DEAD-box RNA helicase eIF4AII through interaction with the CCR4-NOT complex (Meijer et al., 2013).

In human cells, the DEAD box helicase RCK/p54 interacts with Ago proteins in affinity-purified active siRISC or miRISC, and directly interacts with Ago1 and Ago2 *in vivo* (Chu and Rana, 2006). Human RCK/p54 and its yeast ortholog DHH1 facilitate the formation of P-bodies, and are general repressors of translation (Coller and Parker, 2005). Translation repression by miRNA-induced gene silencing requires human RCK/p54 (Chu and Rana, 2006), suggesting that CCR4-NOT in the miRNA-induced silencing complex may recruit RCK/p54 thereby repressing translation of miRNA targets. However, the mechanism of miRNA-mediated translation repression remains largely unclear.

## A NOVEL ROLE OF CCR4-NOT IN PROTEIN QUALITY CONTROL BY TRANSLATION ARREST

The NOT4 subunit of the yeast CCR4-NOT complex is an E3 ubiquitin ligase of the RING family type that catalyzes protein ubiquitination. The RING domain of NOT4 is crucial for its interaction with the E2 ubiquitin ligases, Ubc4 and Ubc5, but not for its interaction with the CCR4-NOT complex. EGD, an ortholog of the nascent associated polypeptide complex (NAC), is the first described substrate of NOT4 in yeast. EGD, which is composed of Egd1 and Egd2, is ubiquitinated by Not4 but its ubiquitination is also affected by the absence of other subunits of the CCR4-NOT complex (Panasenko et al., 2006; Mulder et al., 2007). EGD ubiquitination has been proposed to contribute to NAC association with the ribosome and with the proteasome (Panasenko et al., 2009). Ubc4 interacts with the proteasome in response to translationally damaged proteins (Chuang and Madura, 2005).

**FIGURE 3:**
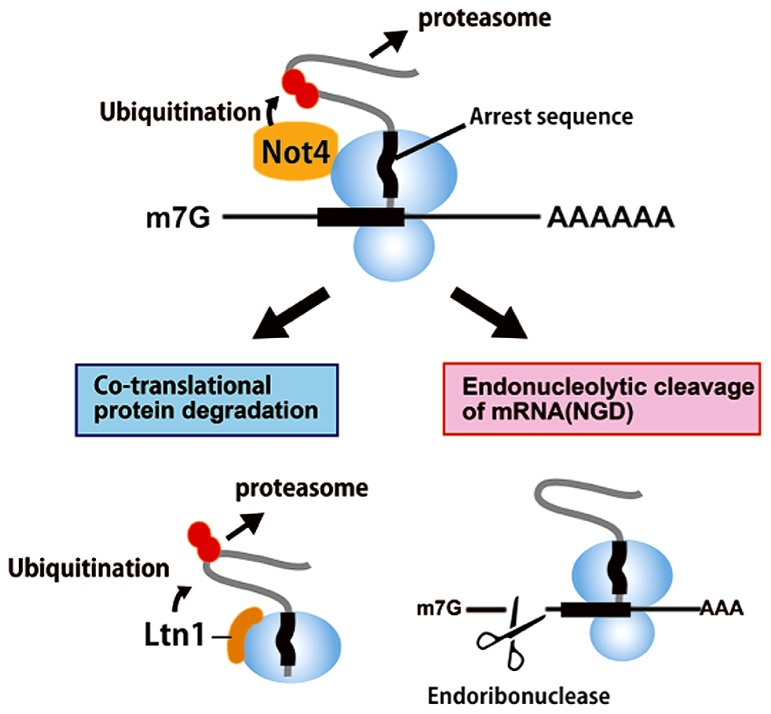
**A novel role of CCR4-NOT in protein quality control by translation arrest**.The NOT4 subunit of the yeast CCR4-NOT complex is an E3 ubiquitin ligase. EGD, an ortholog of the nascent associated polypeptide complex (NAC), is the first described substrate of NOT4 in yeast. In addition of Ltn1, NOT4 is involved in co-translational clearance of aberrant proteins by the proteasome in yeast. The stalling of ribosomes during translational elongation results in co-translational degradation of the arrested protein product by the proteasome (ribosome quality control, RQC) as well as an endonucleolytic cleavage of mRNA no-go decay (NGD). NOT4 synergizes with the important E3 ligase Ltn1 for the degradation of arrest products. Ltn1 has been suggested to recognize the 60S subunit containing peptidyl-tRNA, which arises from the dissociation of the ribosome into 40S and 60S subunits by an unknown mechanism.

NOT4 is not essential for deadenylase activity or for assembly of the CCR4-NOT complex (Bai et al., 1999). However, an recent study suggested that the NOT4 E3 ubiquitin ligase is involved in co-translational protein quality control (Dimitrova et al., 2009; **Figure 3**). The stalling of ribosomes during translational elongation that is induced by a nascent peptide results in co-translational degradation of the arrested protein product by the proteasome (Dimitrova et al., 2009). NOT4 associates with polyribosomes, as revealed by polysome fractionation (Dimitrova et al., 2009; Panasenko and Collart, 2012), and is involved in protein degradation of the translation arrest products produced by poly lysine sequences but not by those of non-stop proteins (Dimitrova et al., 2009). The poly(A) tail is not required for protein degradation of the translation arrest products, indicating that NOT4 is not recruited by binding to the poly(A) tail (Matsuda et al., 2014). Consistent with these data, it was shown that NOT4 plays a role in protein quality control independent of the CCR4 deadenylase (Halter et al., 2014). NOT4 is involved in assembly of the proteasome (Panasenko et al., 2009) and in clearance of aberrant proteins at least in part via the proteasome (Halter et al., 2014).

The E3 ligase Ltn1 plays a crucial role in the degradation of arrest products produced by the translation of poly(A) sequences (Wilson et al., 2007; Bengtson and Joazeiro, 2010) and derived from mRNAs containing arrest-inducing sequences, including polybasic amino acid sequences and rare codons (Matsuda et al., 2014). Ltn1 may recognize peptidyl-tRNA on stalled ribosomes and ubiquitinate this complex for degradation by the proteasome. In contrast, NOT4 may bind to the 80S ribosome that is stalled within the mRNA, but not at the 3′ end of the mRNA. These results are consistent with the ribosome binding specificity of these ligases, as NOT4 is found in the polysome fractions (Dimitrova et al., 2009; Halter et al., 2014) and Ltn1 is mainly distributed in the 60S subunit (Bengtson and Joazeiro, 2010; Brandman et al., 2012). Ltn1 and NOT4 have synergistic effects on the degradation of arrest products, and the poly(A) tail is not required for the degradation of arrest products by these two ligases (Matsuda et al., 2014). Future experiments will elucidate the mechanism by which these two ubiquitin ligases facilitate the proteasomal-degradation of arrest products on stalled ribosomes.

## PERSPECTIVE

The CCR4-NOT complex is a highly conserved gene silencer that is essential for diverse cellular functions. Two nuclease activities of the CCR4-NOT complex play crucial roles in gene regulation at post-transcriptional levels. The CCR4-NOT complex contributes to miRNA-induced gene silencing and functions both as a scaffold for the binding of specific regulators and as a major cellular deadenylase. The precise roles of CCR4-NOT in the stimulation of decapping and translational repression of miRNA targets must be elucidated in order to understand the mechanism of miRNA-induced gene silencing. An E3 ubiquitin ligase activity of the NOT4 subunit also contributes to protein quality control. However, it is still largely unknown how the NOT4 subunit of the CCR4-NOT complex recognizes aberrant nascent peptides or what the precise roles of CCR4-NOT in the protein quality control system induced by translation arrest are. Future experiments will elucidate molecular mechanisms of gene regulation by this complex and the novel roles of CCR4-NOT in diverse cellular functions.

## Conflict of Interest Statement

The authors declare that the research was conducted in the absence of any commercial or financial relationships that could be construed as a potential conflict of interest.
